# An Integrated Neural Framework for Dynamic and Static Face Processing

**DOI:** 10.1038/s41598-018-25405-9

**Published:** 2018-05-04

**Authors:** Michal Bernstein, Yaara Erez, Idan Blank, Galit Yovel

**Affiliations:** 1 Sagol School of Neuroscience, Tel Aviv University, Tel Aviv, 6997801 Israel; 2 MRC Cognition and Brain Sciences Unit, 15 Chaucer Rd, Cambridge, UK; 3 Department of Brain and Cognitive Sciences, Massachusetts Institute of Technology, Cambridge, MA USA; 4 School of Psychological Sciences, Tel Aviv University, Tel Aviv, 6997801 Israel

## Abstract

Faces convey rich information including identity, gender and expression. Current neural models of face processing suggest a dissociation between the processing of invariant facial aspects such as identity and gender, that engage the fusiform face area (FFA) and the processing of changeable aspects, such as expression and eye gaze, that engage the posterior superior temporal sulcus face area (pSTS-FA). Recent studies report a second dissociation within this network such that the pSTS-FA, but not the FFA, shows much stronger response to dynamic than static faces. The aim of the current study was to test a unified model that accounts for these two functional characteristics of the neural face network. In an fMRI experiment, we presented static and dynamic faces while subjects judged an invariant (gender) or a changeable facial aspect (expression). We found that the pSTS-FA was more engaged in processing dynamic than static faces and changeable than invariant aspects, whereas the OFA and FFA showed similar response across all four conditions. These findings support an integrated neural model of face processing in which the ventral areas extract form information from both invariant and changeable facial aspects whereas the dorsal face areas are sensitive to dynamic and changeable facial aspects.

## Introduction

**Figure 1:**
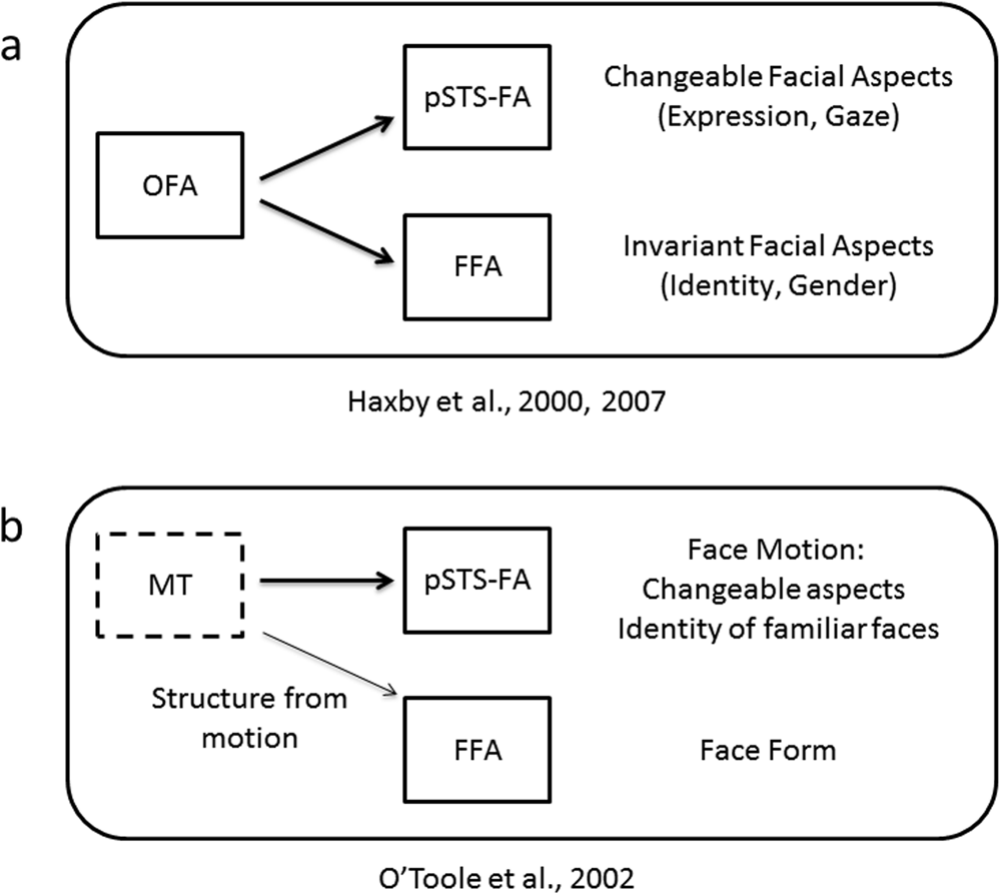
Neural models of face processing. (**a**) The current dominant neural model of face processing^1,4^ includes two pathways: a ventral pathway, including the FFA, processes invariant facial aspects, such as identity and gender, whereas a dorsal pathway, including the pSTS-FA, processes changeable facial aspects such as facial expression and eye gaze. The OFA is involved in early stages of face processing and provides input to both pathways. (**b**) Modifications to this model for dynamic faces^8^: the ventral pathway processes facial form while the dorsal pathway processes facial motion. The motion-selective area MT provides input to the dorsal, motion pathway, and may also send “motionless” structure information to the ventral, form pathway.

Faces generate highly selective responses in high-level visual cortex. In particular, three face-selective areas are typically revealed, the occipital face area (OFA), the fusiform face area (FFA) and the posterior superior temporal sulcus face area (pSTS-FA)^1^. The functional characterization of these areas has been the focus of investigation of numerous studies^2,3^. According to the current dominant model^1,4^ the FFA is primarily engaged with the processing of invariant facial aspects (e.g., identity) whereas the pSTS-FA is primarily engaged with the processing of changeable facial aspects (e.g., expression, see Fig. 1a). Whereas this model is primarily based on studies that presented static faces, recent studies that used dynamic stimuli (i.e. moving faces) revealed that the pSTS-FA and the inferior frontal gyrus face area (IFG-FA), but not the OFA and FFA, show higher response to dynamic (motion) than static (form) faces^5–7^. These findings are consistent with a model suggested by O’toole and colleagues^8^, which highlights the role of the pSTS-FA in the processing of dynamic faces (Fig. 1b).

**Figure 2:**
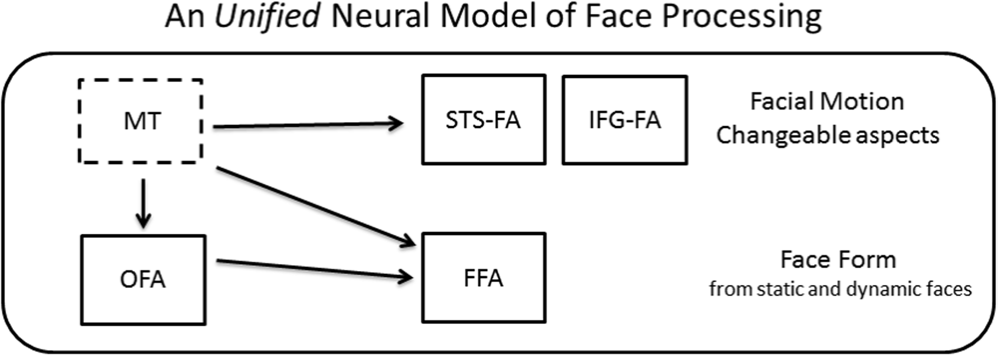
The unified neural model of face processing. The dorsal face areas are engaged in the processing of facial motion and changeable facial aspects, whereas the ventral areas are engaged in the processing of facial form. Area MT sends input to the dorsal areas for motion processing, and to the ventral areas for structure-from-motion analysis.

To integrate these two related functional characterizations of the face network, we have recently proposed a comprehensive neural model of face processing^2,9^ (Fig. 2) according to which the dorsal face areas, including the pSTS-FA and IFG-FA, are engaged in the processing of facial motion and also in the processing of changeable facial aspects. This suggestion is based on behavioral studies showing that expression processing benefits from dynamic information more than identity processing^10–13^. In contrast, the ventral face areas, including the OFA and FFA, are engaged in the processing of facial form, and thus are recruited when processing both changeable and invariant facial aspects for both dynamic and static faces. Additionally, based on previous findings of structural and functional connectivity among the face areas^14–16^ we suggested that the OFA is primarily connected to the FFA but not to the pSTS-FA.

**Figure 3:**
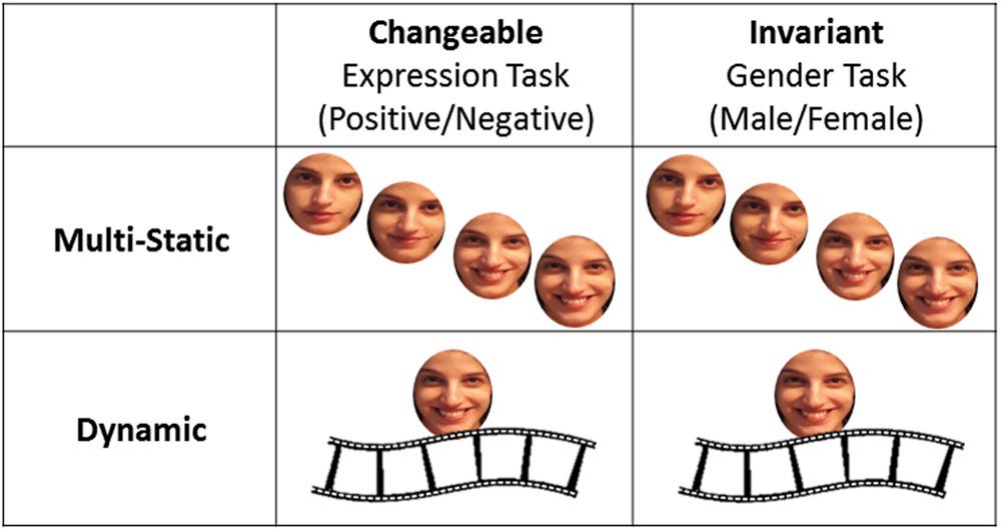
Experimental design and stimuli. Faces were cropped to show only the internal facial features, with no hair or other external facial features. The dynamic conditions included 4 second movie-clips of neutral faces turning happy or disgust. The static conditions included four images from the movie (the first frame of each second) presented for 1 second each in order of appearance in the movie (Version 1) or in a scrambled order (Version 2).

**Figure 4:**
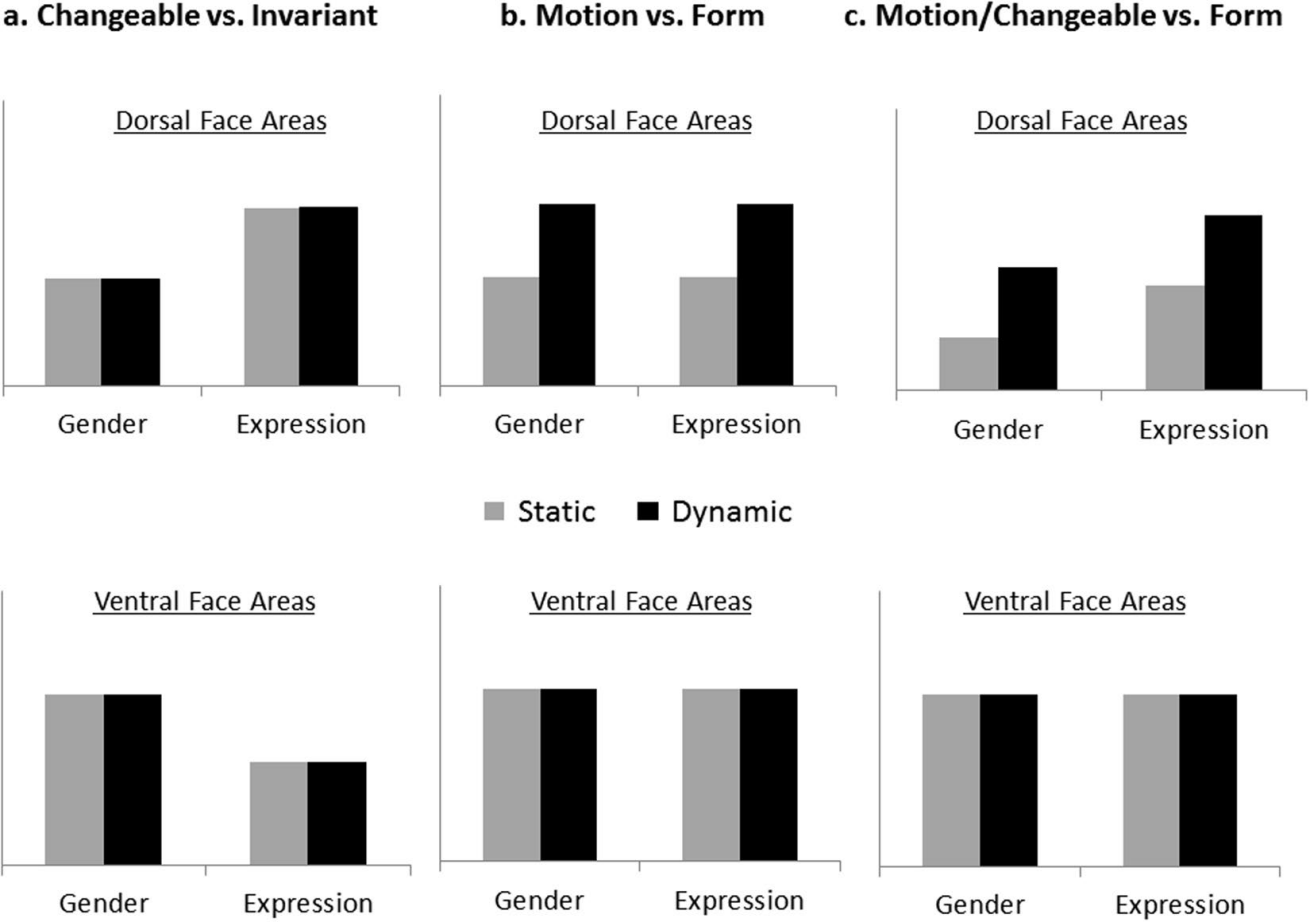
Three alternative predictions. (**a**) The primary division of labor between the dorsal and ventral face areas is to changeable and invariant aspects, as proposed by the current face processing neural model^1,4^. (**b**) The primary division of labor is to motion and form processing. c. An integrated model in which the dorsal face areas are sensitive to both face motion and changeable facial aspects and the ventral face areas extract form information from dynamic and static faces for both invariant and changeable aspects.

Here, we provide the first direct test of this model. We used a factorial design that crossed task requirements for processing either an invariant (i.e., gender) or a changeable facial aspect (i.e., expression) with face stimuli that either contained motion information in them or did not (Fig. 3). We assessed the fMRI responses to these different tasks and stimuli classes in the ventral and dorsal face areas, as well as the motion area, MT. This design allowed us to test the following three hypotheses regarding the division of labor between the dorsal and ventral face-processing pathways (see Fig. 4): (i) if the primary division of labor is to changeable vs. invariant facial aspects (Fig. 4a), then the dorsal face areas would respond strongly during the expression task and the ventral face areas during the gender task, for both static and dynamic faces; (ii) if the primary division of labor is to motion vs. form processing (Fig. 4b), the dorsal face areas would respond to dynamic faces more than static, whereas the ventral face areas would respond similarly to both dynamic and static faces, regardless of the task; (iii) if our model provides an accurate characterization of the face-processing network, the dorsal face area would show a larger response to dynamic than static faces, and also during the expression than the gender task, whereas the ventral face areas would be similarly sensitive to dynamic and static faces and to changeable and invariant facial aspects (Fig. 4c). Finally, to assess the proposed links between the face areas for the processing of dynamic faces, we also examined the functional connectivity among the face areas and with the motion area, MT.

## Results

The experiment included three parts: (i) a functional localizer task to define the areas of the face-processing network; (ii) a functional localizer task to define the motion-selective area MT; and (iii) the main experiment. During the main experiment, dynamic faces (4 second movie-clips) and multi-static faces (4 images from the movie presented for 1 second each) were presented while subjects were instructed to discriminate the face gender (male/female) or expression (positive/negative) alternately (see Fig. 3). We ran two versions of multi-static stimuli on two groups of subjects. In the first version multi-static stimuli were presented in order of their presentation in the movie-clip (n = 18) and in the second version they were presented in a scrambled order to avoid the possible effect of apparent motion (n = 18).

We first analyzed data from the first version of the experiment, in which static images were presented in chronological order. We ran an ANOVA with Hemisphere, Motion (Dynamic, Static) and Task (Expression, Gender) as repeated measures to assess whether there was a significant interaction of the effects of interest with hemisphere. This analysis was conducted separately for each ROI (FFA, OFA, pSTS-FA and MT) to maximize the number of subjects that can be included in the analysis. No interactions were found between any of the factors and Hemisphere (p > 0.1), and therefore for the rest of the analysis, data from the two hemispheres were collapsed using weighted averages based on the volume of each ROI. Because only 5 subjects showed a face-selective response in the right IFG, we report the analysis of data from this region later in a combined analysis of the two versions of the experiment.

**Figure 5:**
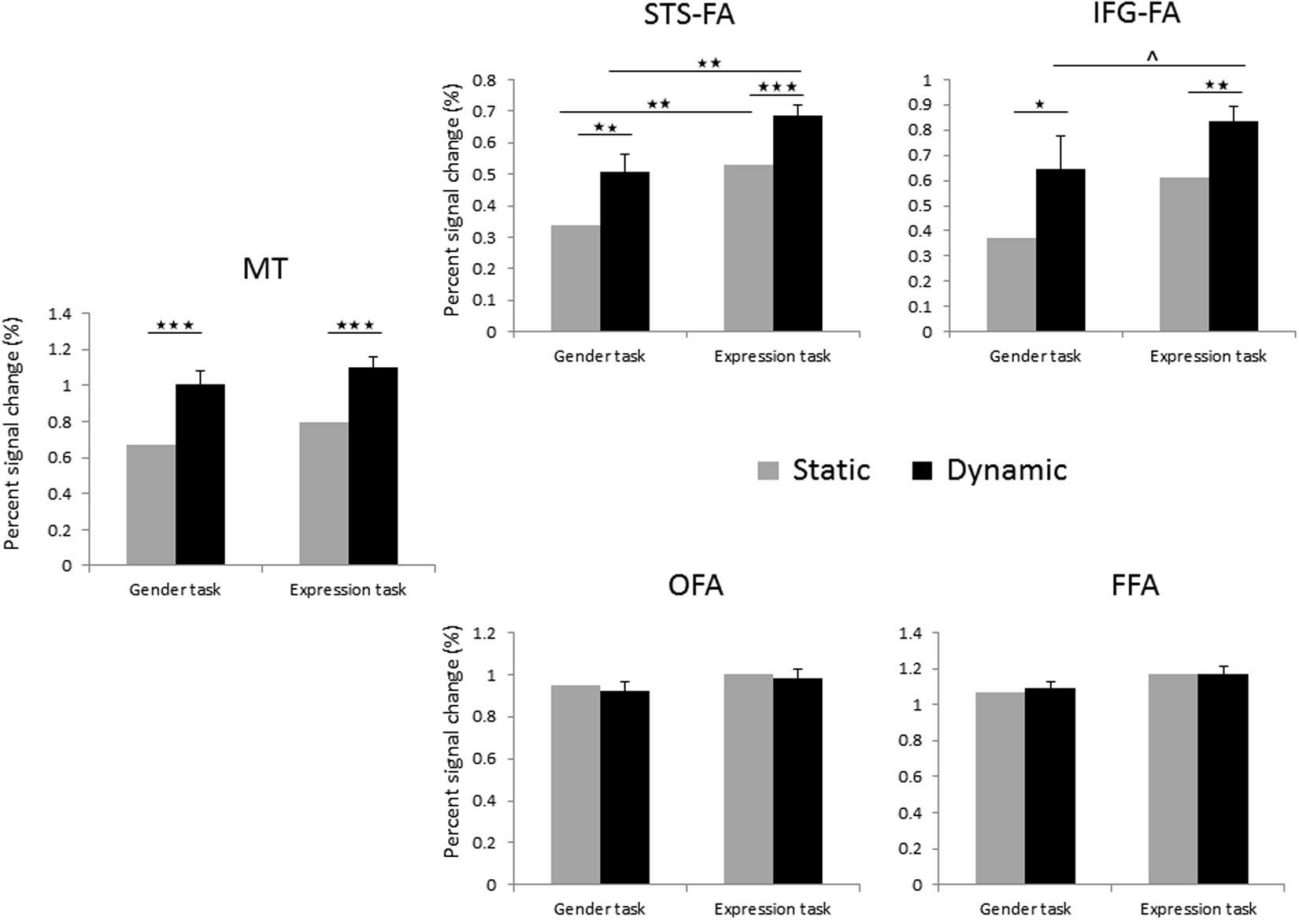
fMRI responses in each ROI including the maximal number of subjects that showed activation in this area (Supplementary Table 3). Error bars indicate the standard error of the mean difference between static and dynamic faces and are shown on one of the bars. *p < 0.05; **p < 0.01; ***p < 0.001.

We next examined the effects of the Motion and Task as repeated measures in each ROI (Fig. 5, Supplementary Table 3). The OFA (n = 14) and FFA (n = 17) showed no effects of Motion (FFA: F(1, 16) < 1, OFA: F(1, 13) < 1) or Task (FFA: F(1, 16) < 1, OFA: F(1, 13) = 2.48, p = 0.13) indicating similar engagement during the changeable and invariant tasks and no sensitivity to face motion. In contrast, the pSTS-FA (n = 17) showed a main effect of Motion (F(1, 16) = 21.19, p = 0.0002, η_p_^2^ = 0.56) indicating higher response to dynamic than static faces, both during the expression task (t(16) = 2.60, p = 0.01) and the gender task (t(16) = 4.71, p = 0.0002). The pSTS-FA also showed a main effect of task (F(1, 16) = 4.75, p = 0.04, η_p_^2^ = 0.22) reflecting higher response during the expression than the gender task. The effect of Task was significant for dynamic faces (t(16) = 2.28, p = 0.03) and marginally significant effect for static faces (t(16) = 1.89, p = 0.07). There was no interaction between Motion and Task (F < 1) indicating that the pSTS-FA is sensitive to both facial properties, motion and changeable aspects, independently.

Finally, we examined the response of the motion area MT to the face stimuli during the two tasks. Area MT (n = 14) showed higher response to the dynamic than the static faces (F(1, 13) = 43.37, p = 0.00001, η_p_^2^ = 0.76), both during the expression task (t(13) = 3.39, p = 0.004) and during the gender task (t(13) = 7.91, p = 0.0001). No further effects or interactions were found.

To assess whether the patterns of response that we found are significantly different between the dorsal and ventral face areas, we combined the data from the pSTS-FA and FFA and added ROI as a within subject factor in the ANOVA (n = 17). This analysis revealed a significant interaction between ROI and Motion (F(1, 16) = 19.23, p = 0.0004, η_p_^2^ = 0.54) indicating the sensitivity of the pSTS-FA, but not the FFA, to facial motion. The interaction of ROI and Task (F(1, 16) = 2.3, p = 0.16, η_p_^2^ = 0.13) was not significant indicating that the preference of the pSTS-FA to the expression task was not significantly different from the FFA.

To compare the pattern of response in the OFA and FFA, ANOVA with Motion, Task and ROI (OFA, FFA) (n = 13) was performed. The analysis did not reveal significant interactions of Motion or Task with ROI (p > 0.5), indicating that OFA and FFA showed similar pattern of response.

Analysis of the second version of the experiment, in which static images were presented in a scrambled order to prevent possible effects of apparent motion, revealed similar findings to those obtained when static images were presented in order (see Supplementary text). We therefore combined data from the two versions of the experiment to examine the effects on a larger sample. We performed a mixed ANOVA with Version as a between subject factor and Task and Motion as within-subject factors. None of the ROIs showed an interaction with Version. The Results of the data combined across the two versions are shown in Fig. 5.

The OFA (n = 31) and FFA (n = 34) showed no effects of Task (OFA: F(1, 29) = 1.41, p = 25, FFA(1, 32) = 2.3, p < 0.13) or Motion (OFA: F(1, 29) < 1, FFA(1, 32) < 1). In contrast, the pSTS-FA (n = 33) showed a main effect of Motion (F(1, 32) = 20.14, p < 0.0001, η_p_^2^ = 0.39) as well as a main effect of Task (F(1, 32) = 11.49, p = 0.002, η_p_^2^ = 0.27) with no interaction between the two factors (F(1, 32) = 0.04, p = 0.83). The response to dynamic faces was larger than static faces both during the expression task (t(32) = 4.31, p = 0.00001) and during the gender task (t(32) = 3.07, p = 0.004). The response during the expression task was larger than during the gender task for both dynamic (t(32) = 3.08, p = 0.004) and static faces (t(32) = 2.94, p = 0.006).

Area MT (n = 30) showed higher response to the dynamic than the static faces (F(1, 28) = 33.58, p = 0.0001, η_p_^2^ = 0.54), both during the expression task (t(29) = 5.65, p = 0.00001) and during the gender task (t(29) = 4.70, p = 0.00001). The effect of Task was marginally significant (F(1, 28) = 3.45, p = 0.07, η_p_^2^ = 0.10), and there was no interaction between Motion and Task (F(1, 28) = 0.25, p = 0.61).

The larger sample allowed us to explore the pattern of response in the IFG-FA which was functionally identified in 11 subjects across the two versions of the experiment. The IFG-FA showed a main effect of Motion (F(1, 9) = 57.19, p < 0.0001, η_p_^2^ = 0.86) indicating a higher response to dynamic than static faces. There was no interaction between Motion and Task indicating that the higher response to dynamic faces was found for both changeable (t(10) = 3.84, p = 0.003) and invariant facial aspects (t(10) = 2.16, p = 0.05). Despite numerically higher response during the expression than the gender task (see Fig. 5), the IFG-FA showed no effect of Task (F(1, 9) = 1.9, p = 0.19).

To compare the results in area MT to those of the pSTS-FA we included ROI (MT, pSTS-FA) as additional within subject factor. This ANOVA revealed a main effect of ROI (F(1, 26) = 15.67, p < 0.0005, η_p_^2^ = 0.37), a main effect of Motion (F(1, 26) = 32.11, p < 0.000006, η_p_^2^ = 0.55), a main effect of Task (F(1, 26) = 7.49, p < 0.01, η_p_^2^ = 0.22) and an interaction of ROI and Motion (F(1, 26) = 25.41, p < 0.00003, η_p_^2^ = 0.49). The interaction of ROI and Task was not significant (F(1, 26) = 2.22, p < 0.14), suggesting that there may be a close link between motion processing and the processing of changeable facial aspects.

Finally, to examine whether the response of the pSTS-FA was significantly different than the response of the FFA on this larger sample we included ROI (FFA, pSTS-FA) as additional within subject factor. An interaction between ROI and Motion F(1, 31) = 31.16, p < 0.0001, η_p_^2^ = 0.50) indicated greater sensitivity to motion than static faces in the pSTS-FA but not the FFA. An interaction between ROI and Task F(1, 31) = 7.68, p < 0.01, η_p_^2^ = 0.20) indicated greater sensitivity during the expression than the gender task in the pSTS-FA but not the FFA. The 3-way interaction of ROI, Motion and Task was not significant (F(1, 31) < 1) indicating that the effect of Task and Motion were additive.

Overall these findings show that the OFA and FFA showed no sensitivity to motion and respond similarly during the expression the gender tasks. The pSTS-FA showed higher response to dynamic than static faces during both the expression and the gender tasks and higher response during the expression than the gender task for both dynamic and static faces. The IFG-FA was also highly responsive to dynamic stimuli but did not show a significantly higher response during the expression than the gender task.

## Functional Connectivity

Results so far suggest functional dissociation between the pSTS-FA and the ventral face areas, OFA and FFA. These findings may be consistent with previous anatomical connectivity^14,15^ and functional correlation^16^ studies that reported stronger structural connections and synchronization between OFA and FFA than with the pSTS-FA. Here, we were interested in replicating these findings as well as examining the functional connectivity between the face areas and the motion area MT. This allowed us to evaluate the model suggested by O’Toole and colleagues according to which area MT is connected with both the pSTS-FA for the processing of dynamic information as well as with the ventral face areas for the extraction of form from motion^8^ (Fig. 1b). Therefore, we examined the functional synchronization between the face areas and MT by correlating their respective signal time-courses recorded during the main task.

**Figure 6:**
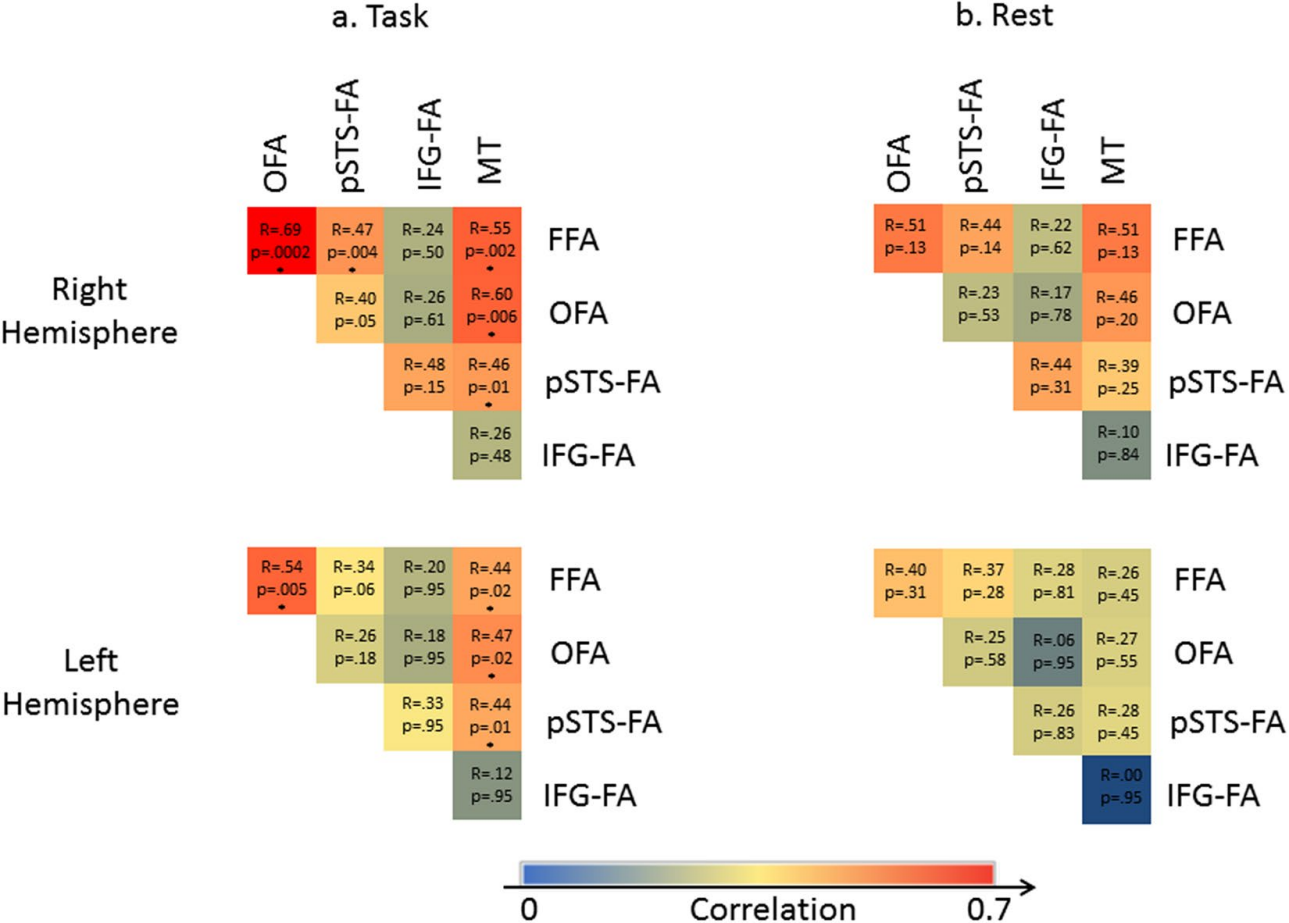
Functional connectivity of the face processing system during task (**a**) and rest (**b**) The color code indicates the level of correlation calculated between each pair of regions in each subject and then averaged across subjects. *p < 0.05.

To assess the relative strength of functional connections between the different ROIs, the correlations were converted to Fisher Z scores and a repeated-measures ANOVA with ROI pairs (MT-OFA, MT-FFA, MT-pSTS-FA, OFA-FFA, OFA-pSTS-FA, FFA-pSTS-FA) and Hemisphere was performed. The ANOVA revealed a significant effect of ROI pairs (F(5, 60) = 12.60, p < 10^−8^, η_p_^2^ = 0.51) and a significant interaction of ROI pairs and Hemisphere (F(5, 60) = 3.18, p = 0.01, η_p_^2^ = 0.20), therefore correlations are reported separately for the right and left ROIs. As shown in Fig. 6a, the OFA was strongly correlated with the FFA but less correlated with the pSTS-FA (right: t(22) = 7.88, p < 10^−7^; left: t(23) = 7.46, p < 10^−9^). Area MT was similarly correlated with the FFA, OFA and pSTS-FA (p > 0.3).

The IFG-FA was not included in this ANOVA because of the small number of participants showing activation in this region, especially on the left side. However, as Fig. 6 shows, the right IFG-FA was more correlated with the pSTS-FA than with the ventral face areas (right FFA: t(9) = 7.37, p < 10^−5^; right OFA: t(5) = 2.51, p = 0.05), consistent with the univariate findings showing sensitivity to motion in the pSTS-FA and IFG-FA but not in the ventral areas.

To ensure that these correlation patterns are independent of task, we examined the functional synchronization across the face-processing network during “rest”. Figure 6b presents matrices of pairwise correlations between the face-selective areas and area MT, averaged across subjects. An ANOVA with ROI pairs and Hemisphere as repeated measures and Type of processing (Face processing task, Rest) as a between-subjects factor revealed a significant effect of ROI pairs (F(5, 85) = 9.31, p < 10^−7^, η_p_^2^ = 0.35) but no interactions with the Type of processing (p > 0.4), indicating that the functional correlation patterns, although weaker than in the resting state experiment (see also^16^), were similar across rest and task. Thus, the resting state functional correlations showed similar patterns of stronger synchronization between the ventral areas FFA and OFA, weaker synchronization between the OFA and the pSTS-FA, and synchronization of all three regions with area MT.

## Discussion

The goal of the current study was to integrate two major functional characteristics of the ventral and dorsal face areas, their sensitivity to changeable vs. invariant facial aspects^1^ and their different sensitivity to motion^6–8^. Our results show that the dorsal face areas, pSTS-FA and IFG-FA responded more strongly to dynamic than to static faces^5,6^ regardless of whether participants performed an expression or a gender task. The pSTS-FA also responded more strongly during the expression than the gender task regardless of whether faces were moving or not. In contrast, the ventral face areas, FFA and OFA, were responsive to faces regardless of whether they were moving or not^5,6^ and whether subjects categorize invariant or changeable facial aspects^9^ (Fig. 5). These findings critically constrain the relationship between the processing of changeable facial aspects and the processing of facial motion. First, the processing of motion cannot be reduced to the processing of changeable aspects: even when the task requires categorizing invariant aspects, the dorsal face areas are still engaged more strongly in processing dynamic vs. static faces. Thus, this finding is inconsistent with a model that dissociates the processing of changeable and invariant face aspects in the dorsal and ventral face pathways, respectively^1,4^ (Fig. 4a). Second, the processing of changeable aspects cannot be fully accounted for by the processing of facial motion: even in the absence of such motion, the pSTS-FA is still engaged more strongly by processing changeable vs. invariant aspects. More generally, whereas the primary division between the dorsal and ventral face areas appears to separate face motion from form^7,8^ (Fig. 4b), this dissociation alone cannot exclusively account for the functional differences between the two face pathways.

Still, processing dynamic information and processing changeable aspects are not entirely dissociated and appear to be integrated within the face-processing network: regions within this network are either sensitive to both (pSTS-FA) or to neither (OFA and FFA). Specifically, we suggest that the preferential response to changeable facial aspects in the pSTS-FA may be due to their dynamic nature, given that the processing of changeable aspects such as expression relies on dynamic changes of facial structure over time. The association between changeable facial aspects and motion is supported by a large body of behavioral evidence showing a dynamic advantage for expression recognition^10^, contrasting with a less conclusive dynamic advantage for identity recognition^13^.

A previous study by Johnston and colleagues^17^, employing a similar design in which expression and gender tasks were performed on dynamic and static faces, has reported stronger response in the STS to the dynamic than to the static faces, and during the expression than the gender task, consistent with our findings. However, in this study the fusiform gyrus was also found to respond more strongly to dynamic than static faces, and during the expression than the gender task. A stronger response in the fusiform gyrus to dynamic than static faces is inconsistent with our results as well as with previous studies contrasting dynamic and static stimuli^5,6^. This contradiction may be explained by the type of dynamic stimuli used in these studies: while Fox and colleagues^5^, Pitcher and colleagues^6^ and the present study, used videos of real-life moving faces for the dynamic conditions, Johnston and colleagues^17^ used computer-generated morphs between neutral and expressive faces. Such morphs were previously shown to activate the fusiform gyrus^18–21^. However, the morphed stimuli may not resemble natural dynamic expression and may actually emphasize form changes rather than dynamic aspects of naturally folding facial expressions.

The integrated neural framework of face processing that is supported by our findings^2,9^ (Fig. 2) is consistent with several reports that have been recently published and may not easily fit with current models. First, whereas the classic models of face recognition^1,22^ proposed parallel and independent routes for invariant and changeable facial aspects, recent studies have found that the processing of identity and expression may be mediated by the same system rather than being fully separated^12^. A common mechanism for the processing of changeable and invariant aspects is also supported by computational and neuropsychological data^23^, and by fMR-adaptation studies showing sensitivity of the FFA to both facial expression and identity^24–27^. These findings suggest that the primary division of labor between the ventral and dorsal face areas may not be the division to invariant and changeable aspects.

Second, whereas the current dominant model^1,4^ posits that the OFA is a source of input to both the ventral and the dorsal face pathways, recent connectivity studies^14,15^ have instead revealed that the OFA may be connected to the FFA more than it is to the pSTS-FA. In line with previous work^16^, we found stronger functional correlation between the OFA and FFA and weaker correlation with the pSTS-FA, both during face processing tasks and at rest. These findings are in line with the similar response profiles to face stimuli in the FFA and OFA, which differ from the response profile of the pSTS-FA. O’Toole and colleagues^8^ have suggested that the pSTS-FA may receive input from area MT, which is consistent with the finding that both these regions are engaged in motion processing. Indeed, we found functional correlations between area MT and the pSTS-FA, although area MT was also correlated with the FFA and OFA. We suggest that these latter correlations between area MT and the ventral face areas may allow for “form-from-motion” analysis, as proposed by O’Toole and colleagues^8^. According to their model, structural information is extracted from dynamic faces in area MT and sent back to the ventral stream as ‘motionless’ form information for structural analysis. Importantly, this is the first study to our knowledge that specifically examined functional coupling between area MT and the face selective areas, thereby providing empirical support for the connections between them as proposed by O’Toole and colleagues. It is noteworthy that the face areas in the current study were defined using a dynamic face localizer, which may result in higher functional correlations between all face areas and area MT relative to areas defined by static images. However, given the large overlap between the ventral areas that are identified using static and dynamic functional localizers^5^, the patterns of their functional synchronization with MT are likely to be similar. Alternatively, the current functional correlations specifically reflect association of the motion area with the processing of dynamic faces by the face network.

In addition to the face-selective areas discussed above, recent studies that used a dynamic face localizer have reported a prefrontal face-selective area, typically located in the IFG^5,6^. The functional profile of the IFG face area (IFG-FA) has been only scarcely studied^28^, possibly due to its relatively low response to the prevalently used static face stimuli. Here, we functionally defined this region with a dynamic face localizer and still found it only in less than 1/3 of the subjects we scanned. Similar to the pSTS-FA, the IFG-FA showed a stronger response to dynamic than static faces. Unlike the pSTS-FA, the response of the IFG-FA was numerically larger during the expression than the gender task (see Fig. 5), but the difference was not statistically significant possibly due to the low power of the analysis. Nevertheless, recent studies did indicate that the IFG-FA may play a role in the processing of eye-gaze information^29^. However, the IFG-FA was shown to be involved also in the representation of face identity^30^. Thus, the precise functional role of the IFG-FA remains to be elucidated in future studies.

An additional anterior face-selective area that has been revealed in fMRI studies of face processing, is located in the ventral anterior temporal cortex (ATL-FA, for a review see^31^). The current study did not test the role of the ATL-FA in the processing of changeable and invariant aspects of dynamic and static faces, because the low signal-to-noise ratio in this part of the brain makes it challenging to localize this area with the type of localizer scans that we used. The few studies that were able to reveal face-selective responses in the ATL of most of their participants have done so using a functional localizer that involved familiar and/or expressive faces^16,32^ or using coronal rather than horizontal scanning^33^. However, the fact that the dynamic face localizer that we used in the current study did not reveal strong responses in the ventral anterior temporal cortex, may imply that the ATL-FA is part of the ventral face stream and is not particularly sensitive to dynamic face information. Furthermore, a recent monkey electrophysiology study of the face-selective area AM, which is considered the monkey-homologue of the ATL-FA^34^, showed its sensitivity to shape and appearance of facial features^35^. These findings are consistent with the role of this area in the processing of form information from faces. Further research directed at the ATL-FA is needed to address its role in our proposed face model.

Finally, the dorsal face areas may be part of a more general system for the processing of the whole dynamic person^36^. In particular, the STS has been shown to be activated by dynamic bodies such as point light displays^37–39^, dynamic full light bodies^40^ as well as voices^41,42^. Whereas the model suggested here focuses on the processing of dynamic faces, it is noteworthy that the STS is suggested to be a neural hub for multi-modal representation of the whole dynamic person^36^.

In summary, we report a comprehensive and systematic characterization of two central functional characteristics of the face processing network that have so far studied separately - sensitivity to changeable and invariant facial aspects and to dynamic and static facial stimuli. Our revised framework goes beyond previous accounts in that it shows that (i) the dorsal face area, the pSTS-FA is sensitive to motion and to changeable facial aspects (ii) the ventral face areas, the OFA and FFA, are similarly sensitive to changeable and invariant facial aspects and are insensitive to motion, and (iii) the motion area MT is functionally associated with both the ventral and dorsal dynamic face areas. This integrated model provides an ecologically motivated framework for the functional architecture of the face-processing network, one that can better accommodate the dynamic faces we encounter in the real world.

## Methods

### Participants

Forty-one healthy volunteers (19 men, ages 18–35) with normal or corrected-to-normal vision participated in the experiment. Three participants were excluded due to excessive head movements and another two participants were excluded due to pathological findings, leaving a total of thirty-six participants in the final analysis. Participants participated in one of two versions of the experiment: in the first group (N = 18) multi-static stimuli were presented in order of appearance in the movies and in the second group (N = 18) they were presented in a scrambled order (see details below). In addition, 12 healthy volunteers (4 men, ages 21–31) participated in an additional experiment that included the face and motion localizers and a resting state scan (see Supplementary text). Out of the total 48 subjects included in the study (36 for the main task analysis and additional 12 for the resting-state functional connectivity analysis), 41 were right-handed. Participants were paid $15/hr. All participants provided written informed consent to participate in the study, which was approved by the ethics committees of the Sheba Medical Center and Tel Aviv University, and performed in accordance with relevant guidelines and regulations.

### Stimuli

The experiment included three parts: a functional dynamic face localizer to localize the face-selective areas (see for example ref.^5^), a functional motion localizer to localize the motion-selective area MT (see for example ref.^43^), and the main experiment. For details on the stimuli and design of the functional localizers please refer to the supplementary text.

Stimuli for the main experiment were taken from the database of moving and static faces collected at the Vision Lab at UTD^44^. All stimuli were cropped to show only the face area, with no hair or other external facial features (see Fig. 3 and Supplementary Figure 1 for example stimuli). The main experiment included four conditions in a factorial design that crossed stimulus class (dynamic or static) and categorization task (expression or gender): Dynamic faces-Expression task, Static faces-Expression task, Dynamic faces-Gender task, and Static faces-Gender task. In the two dynamic conditions, 4 s movie-clips (24 frames per second) were presented. In the two static conditions, four images from the movie (the first frame of each second) were presented for 1 second each. In the first version of the experiment the four images were presented in their chronological order of appearance within the movie. Such multi-static images provide richer information than one static image and therefore better match the dynamic stimuli (see for example ref.^45^). To minimize the possible effect of apparent motion, in the second version of the experiment the same sets of four images were presented in a randomly scrambled order. 48 different identities were used as stimuli, half females and half males. Twenty-seven of the movies (12 females, 15 males) showed a face transitioning from a neutral expression to a happy (positive) expression, and the remaining 21 movies showed a face transitioning from a neutral expression to a disgust (negative) expression. Importantly, the same 48 identities were used in all four experimental conditions, but the stimuli were counterbalanced across subjects such that each subject saw a given identity in only one of the four conditions.

### Apparatus and Procedure

fMRI data were acquired in a 3 T Siemens MAGNETOM Prisma MRI scanner, using a 64-channel head coil. Echo-planar volumes were acquired with the following parameters: repetition time (TR) = 2 s, echo time = 35 ms, flip angel = 90°, 31 slices per TR, slice thickness = 3 mm, field of view = 20 cm and 96 × 96 matrix, resulting in a voxel size of 2.1 × 2.1 × 3 mm. Stimuli were presented with Matlab Psychtoolbox^46^ and displayed on a 40” high definition LCD screen (NordicNeuroLab) viewed by the participants through a mirror located in the scanner. Anatomical MPRAGE images were collected with 1 × 1 × 1 mm resolution, echo time = 2.6 ms, TR = 1.75 s.

The main experiment included four runs. Each run included 21 blocks: 5 baseline fixation blocks and 4 blocks for each of the four experimental conditions: Dynamic/Static × Expression/Gender. Each block lasted 16 seconds and included 3 trials. Each trial consisted of a 4 s stimulus (a 4s movie-clip in the dynamic conditions; four images from the movie presented for 1s each in the static conditions) and an inter-trial-interval (ITI) of 1.33 seconds. The order of the experimental conditions was counterbalanced within and across runs. Subjects’ task was to discriminate the gender (male/female) or expression (positive/negative) of each face. During each fixation block, instructions appeared on the screen to inform the subject of the task to be performed on the next four blocks. Subjects responded by pressing one of two response buttons, either while the stimulus was presented or during the ISI.

### Data Analysis

#### Behavioral Data Analysis

We calculated accuracy (percentage of correct responses) and response times for correct responses for each of the four experimental conditions separately. For the behavioral results see the Supplementary text and Supplementary Table 2.

#### fMRI Data Analysis

fMRI analysis was performed using statistical parametric mapping (SPM8^47^). The first three volumes in each run were acquired during a blank screen display and were discarded from the analysis as “dummy scans”. The data were then preprocessed using slice timing correction and realignment to the first slice of each run. Spatial smoothing was performed for the localizer data only (5 mm). A GLM was run with separate regressors for each run and for each condition.

#### ROI Analysis

Based on the functional localizers data, face- and motion-selective voxels were defined using contrast *t*-maps to assure their functional specificity^5,6^. For details on ROI localization please refer to the Supplementary text and Supplementary Table 1.

#### Main Experiment

BOLD signal time courses for each of the four experimental conditions were extracted from each ROI using the MarsBaR ROI toolbox of SPM^48^. The dependent measure was an average of the BOLD signal values in TRs 3–8. Statistical analysis was performed with Statistica 13. The effects of Task and Motion were examined separately in each of the 5 ROIs (OFA, FFA, STS-FA, IFG, MT) and we therefore used a Bonferroni corrected p-value of 0.05/5 = 0.01.

In addition to the univariate analysis, we also conducted a multivariate analysis using split-half correlations between the neural patterns elicited by the four experimental conditions. The methods and results of this analysis are reported in the Supplementary text. Finally, we performed functional connectivity analysis to examine the correlations between time-courses in the face- and motion-selective ROIs during task and rest. For detailed methods of this analysis see Supplementary text.

### Data availability

The datasets generated during and/or analyzed during the current study are available from the corresponding author on reasonable request.

## Electronic supplementary material

Supplementary material
