# Supplementary material for: An Integrated Neural Framework for Dynamic and Static Face Processing
Source: Sci Rep. 2018 May 4;8:7036. doi: 10.1038/s41598-018-25405-9 (PMC5935689; doi:10.1038/s41598-018-25405-9)
Supplement: Supplementary file 1 — Supplementary material [file 41598_2018_25405_MOESM1_ESM.docx]

An Integrated Neural Framework for Dynamic and Static Face Processing

Michal Bernstein, Yaara Erez, Idan Blank & Galit Yovel

Supplementary Materials

Methods

**Localization of dynamic face areas and motion area MT**

Functional face localizer: Stimuli of the face localizer were dynamic (1s movie-clips) faces and objects (e.g., a ball, a fan). The object stimuli were provided by Fox and colleagues^1^. The face stimuli were filmed in our lab and included 16 different faces which moved their face/head by talking or making various facial expressions or mouth movements.

The functional face localizer included two runs. Each run included blocks of dynamic faces and objects. Each block lasted 16 seconds and included 16 stimuli. Category block order was counterbalanced within and across runs. Each localizer run consisted of eight blocks for each category and five blocks of a baseline fixation point resulting in a total of 21 blocks (336s). To maintain vigilance, participants were instructed to press a response box button whenever two identical video-clips appeared consecutively (a 1-back task). This happened twice per block in a random location.

Functional motion localizer: Stimuli of the MT localizer were white dots moving on a black background, and static shots of these dots^2^.

The MT functional localizer included two runs. Each run included blocks of dynamic and static dots. Each block lasted 16s. Category block order was counterbalanced within and across runs. Each localizer run consisted of eight blocks for each category and five blocks of a baseline fixation point resulting in a total of 21 blocks (336s). Participants were instructed to passively view the dots. The runs of the localizers and the main experiment were interleaved.

For both the face and the MT functional localizer data, category-selective voxels were defined using contrast *t*-maps to assure their functional specificity. Face-selective areas in the fusiform gyrus (FFA), the lateral occipital cortex (OFA), the posterior superior temporal sulcus (pSTS-FA) and the inferior frontal gyrus face area (IFG-FA) were defined based on the dynamic faces > dynamic objects contrast *t*-map (p < .00001, uncorrected). The motion-selective area MT was defined based on the dynamic dots > static dots contrast *t*-map (p < .00001, uncorrected). Because most subjects showed a relatively small MT region using this contrast or no activation at all (n=10), for those subjects who did not show activation we lowered the statistical threshold to p=.01 and used the MarsBaR 'Draw ROI' option^3^ to define a spherical ROI with a radius of 6mm centered at the peak of the contrast map (average MNI coordinates: right MT (40,-71,12); left MT (-41,-73,9), consistent with previous reports, e.g. ref.^4^.

**Multivariate Analysis**

For the multivariate analysis, we used the β estimates that were created from the GLM used for the univariate analysis (see above). Similarities between voxel-wise patterns of effect-sizes (β estimates) were computed using split-half correlations for each ROI, condition, and run of the main experiment. To allow for the comparison across participants and ROIs, we re-defined ROIs with a fixed-size based on the 60 voxels with the highest localizer contrast *t*-values in each area. ROIs with a smaller number of voxels were excluded from the analysis. This was done to avoid dimensionality effects on classifier performance and prevent potential over fitting^5^, while ensuring a sufficient amount of data for effective classification. However, to assure the robustness of the results, similar analyses were also conducted across a range of ROI sizes (30–80 voxels).

Voxel-wise similarity patterns across conditions were computed as follows: first, for each voxel, the mean β value across all conditions was subtracted from each condition-specific β value. This procedure was performed for each run separately. For each condition, the resulting voxel-wise pattern was then averaged across half of the data (two runs) and correlated with the average pattern that was computed based on the other half of the data. These correlations were computed based on every possible split-half of the data and were finally averaged across split-halves to obtain the similarity measure between the two conditions. This procedure was done for each pair of conditions, resulting in a pair-wise correlation matrix reflecting the degree of similarity between the neural patterns evoked by the four experimental conditions.

Next, we used these pair-wise correlations to compute ‘within’ and ‘between’ similarity measures. Specifically, to examine whether a given ROI carries information about Motion, the 'within' correlation was computed as the average of the correlation between Dynamic faces-Gender task and Dynamic faces-Expression task, and the correlation between Static faces-Gender task and Static faces-Expression task; and the 'between' correlation was computed as the average of the correlation between Dynamic faces-Gender task and Static faces-Expression task, and the correlation between Static faces-Gender task and Dynamic faces-Expression task. To examine whether a given ROI carries information about Task, the ‘within’ correlation was computed as the average of the correlation between Gender task-Dynamic faces and Gender task-Static faces, and the correlation between Expression task-Dynamic faces and Expression task-Static faces; and the ‘between’ correlation was computed as the average of the correlation between Gender task-Dynamic faces and Expression task-Static faces, and the correlation between Gender task-Static faces and Expression task-Dynamic faces.

**Functional Connectivity Analysis**

Participants were scanned with the face and motion functional localizers followed by a resting-state scan. The resting-state scan lasted 10 minutes during which they were instructed to close their eyes and let their mind wander but avoid falling asleep. Importantly, this was a new group of participants that did not undergo the main experiment scans.

The fMRI data acquired during performance of the face processing tasks and during “rest” were preprocessed using the CONN toolbox^6^ to remove noise originating from non-neuronal sources, following the preprocessing procedures described in Blank et al.^7^: First, white matter and CSF voxels were identified based on segmentation of the anatomical image. The first five temporal principal components of the BOLD signal time course extracted from the white matter and CSF were regressed out of each voxel’s time course. The first six principal components of the six motion parameters estimated during motion correction were also regressed out, as well as their first time derivative. Second, the residual signal was bandpass filtered (0.008–0.09 Hz) to preserve only low-frequency signal fluctuations. Following these preprocessing steps, we averaged the BOLD signal time course across all voxels in each ROI. For each pair of ROIs, Pearson’s moment correlation coefficient was then computed between their respective time courses, and tested for statistical significance.

Results

**Localization of dynamic face areas and motion area MT**

Supplementary Table 1 presents the average volume of each ROI (number of voxels) across participants and the number of participants in whom they could be defined. Importantly, the response of area MT to the dynamic faces and objects that were used in our dynamic face localizer was similar indicating no difference in the amount of motion presented in the two categories (see Supplementary Figure 2).

Supplementary Figure 3 shows face-selective and motion areas in a several representative subjects. Consistent with previous studies, dynamic faces generated a much larger response in the pSTS-FA than in the FFA (see also Supplementary Table 1). In contrast to most studies that have used static faces and reveal relatively smaller activation in the pSTS-FA^8^, the volume of the pSTS-FA was more than twice larger than the FFA and in most subjects extended inferiorly to the lateral occipital sulcus (LOS).

**Version 1 – Multi-static in chronological order**

Behavioral Results

The behavioral data analysis is based on the results of 12 out of our 18 subjects, as the responses of 6 subjects were not recorded due to technical issues.

*Accuracy*: We calculated the percentage of correct responses during the gender and expression tasks for the dynamic and the static faces (Supplementary Table 2). A two-way ANOVA with Motion (Dynamic, Static) and Task (Expression, Gender) as within-subject factors revealed a main effect of Task (F(1,11)=5.16, p=.04, η_p_^2^=.32), indicating better performance for the gender than the expression task. No other effects or interactions were found.

*Reaction Time*: Prior to statistical analysis RT values were converted to log(RT). A two-way ANOVA with Motion (Dynamic, Static) and Task (Expression, Gender) as within-subject factors on reaction time data revealed a main effect of Task (F(1,11)=15.91, p=.002, η_p_^2^=.59) due to faster reaction time on the gender than the expression task and a Task by Motion interaction (F(1,11)=5.28, p=.04, η_p_^2^=.32) (see Supplementary Table 2).

Overall behavioral results show no motion advantage in the expression or gender tasks. Thus, the current task did not replicate the motion advantage typically found for expression tasks. We will address this issue in the second version of this study, below.

Multivariate Analysis Results

Univariate analysis did not reveal effects of motion and task in the ventral face areas, the OFA and FFA (see main text). To further assess if information about motion and task is represented in each of the face areas we examined the multi-voxel patterns as a function of motion and categorization task. Split-half correlations between voxel-wise effect sizes were used to assess the degree of similarity between the neural response patterns to the four experimental conditions, within each of the four ROIs. To examine whether a given ROI carries information about Motion, we compared two kinds of correlations: the correlations within presentation type (i.e. correlating data obtained for different dynamic faces, or data obtained for different static faces); to the correlations between presentation types (i.e. correlating data obtained for dynamic faces with data obtained for static faces). If a given brain region is sensitive to motion, the neural patterns generated by two dynamic conditions or by two static conditions should be more similar than the neural patterns generated by a dynamic condition and a static condition. Thus, comparing the correlations within and between dynamic and static stimuli gives a measure of the sensitivity to motion^9,10^.

We first examined these multi-voxel patterns for ROIs of a fixed volume (60 voxels). Higher correlation within presentation type than between presentation types (Dynamic/Static) was found in the pSTS-FA (t(17)=6.70, p=.000004) and in area MT (t(12)=3.67, p=.003) but not in the ventral face areas, the OFA and FFA (p>.3), further supporting the sensitivity to motion in the dorsal but not the ventral face areas, as shown in the univariate results above. The same analysis was conducted using varying sizes of ROIs (30–80 voxels) to verify that the effect is not limited to a specific ROI size. The pSTS-FA and area MT showed higher correlation within than between presentation types across the range of ROI sizes (pSTS-FA: p<.0004 for all ROI sizes; MT: p<.02 for 30, 40, 50 and 60 voxels, p<.08 for 70 and 80 voxels).

To examine whether a given ROI carries information about Task, we compared the correlations within task the expression and gender tasks to the correlations between the two tasks (see Methods). None of the ROIs showed higher correlation within than between tasks (p>.2). Thus, the sensitivity to changeable facial aspects in the pSTS-FA that was found in the univariate analysis is not apparent in the multi-voxel pattern of the neural response of these areas.

**Version 2 – Multi-static in scrambled order**

The experiment included three parts: a functional localizer for the face-selective areas, a functional localizer for the motion-selective area MT, and the main experiment. Stimuli of the main experiment were the same as in Version 1, with only one difference: in the static condition the same four images that were presented in Version 1 in the order of appearance in the movie that they were taken from, were presented in Version 2 in a randomly scrambled order. Stimuli of the face functional localizer were exactly as in Version 1.

We also changed the stimuli we used to define MT because the moving dots did not generate strong enough activations. In Version 2 stimuli of the MT functional localizer were moving concentric grayscale rings, and static shots of these rings (e.g., ref.^11^).

Procedure and analysis were similar to Version 1 (see main text). The only difference was that subjects were asked to respond once they made a decision, which resulted in shorter reaction times.

Behavioral Results

*Accuracy*: We calculated the percentage of correct responses during the gender and expression tasks for the dynamic and the static faces. Supplementary Table 2 summarizes the mean and standard deviation for the different conditions. A two-way ANOVA with Motion (Dynamic, Static) and Task (Expression, Gender) as within-subject factors revealed a main effect of Motion (F(1,17)=6.36, p=.02, η_p_^2^=.27 ), a main effect of Task (F(1,17)=5.44, p=.03, η_p_^2^=.24), and an interaction between the two factors (F(1,17)=4.51, p=.04, η_p_^2^=.21), indicating that expression recognition was more accurate for dynamic than static faces (t(17)=3.02, p=.007), while in the gender task performance was similar for the dynamic and static faces (p>.3). Thus, the scrambling of the static images resulted in the motion advantage for expression recognition, in line with previous studies that compared expression categorization between dynamic stimuli and one static image^12^. As seen below, despite these differences in performance across the two versions of the experiment, they both yielded similar pattern of fMRI findings.

*Reaction Time*: A two-way ANOVA with Motion (Dynamic, Static) and Task (Expression, Gender) as within-subject factors on reaction time revealed a main effect of task due to faster reaction times in the gender than the expression task (F(1,17)=53.02, p=.0001, η_p_^2^=.75) and no effect of Motion nor interaction.

Univariate Analysis Results

We first assessed for each ROI (FFA, OFA, pSTS-FA, and MT) whether there was a significant interaction of the effects of interest with hemisphere. Because we found no interaction of any of the factors of interest with Hemisphere (p > .1), data from the two hemispheres were averaged using weighted averages based on the volume of each ROI. Supplementary Table 3 shows the percent signal change in each ROI for the four different conditions.

We then assessed the effect of Task and Motion in each ROI using a repeated measures ANOVA. The FFA (n = 17) and OFA (n=17) showed no effect of Task (FFA: F(1,16) < 1, OFA: F(1,16) < 1) or Motion (FFA:F(1,16) < 1, OFA: F(1,16) < 1). In contrast, the pSTS-FA (n = 16) showed a main effect of motion (F(1,15) = 7.36, p < .01, η_p_^2^=.33) due to higher response to dynamic than static faces and a main effect of Task (F(1,15) = 7.22, p < .02, η_p_^2^=.32) indicating higher response during the expression than the gender task. The interaction was not significant. The larger response during the expression task was found for both static (t(15)=2.25, p=.03) and dynamic faces (t(15)=2.03, p=.05). Finally, area MT (n = 16) showed a main effect of Motion (F(1,15) = 13.33, p < .005, η_p_^2^=.47) due to higher response to dynamic than static faces but no effect of Task or an interaction between Task and Motion. These findings replicate results of Version 1 indicating that the response of the face and motion areas to dynamic and static faces during a changeable and an invariant task is similar regardless of the order in which the four static face stimuli were presented and the differences in behavioral performance that the two tasks yielded.

Multivariate Analysis Results

Split-half correlations were used to assess the degree of similarity between the neural patterns generated by the four experimental conditions, and correlations within vs. between presentation type or task were examined as a measure of sensitivity to motion or task, respectively. Higher correlation within presentation type than between presentation types was found in the pSTS-FA (t(15)=3.41, p=.003) and in area MT (t(12)=3.01, p=.01) but not in the OFA and FFA (p>.2). The pSTS-FA and area MT showed higher correlation within than between presentation types across a range of ROI sizes (30-80 voxels) (pSTS-FA: p<.01 for all ROI sizes; MT: p<.02 for all ROI sizes). None of the ROIs showed higher correlation within than between tasks (p>.15), indicating that they did not carry information about the task that was being performed.

A combined analysis of the multivariate data across the two versions of the experiment was first examined for ROIs of a fixed volume (60 voxels). Results of data combined from the two versions are shown in Supplementary Figure 4. Higher correlation within presentation type (Dynamic/Static) than between presentation types was found in the pSTS-FA (t(33)=4.3, p=.0001), IFG-FA (t(7)=3.61, p=.008) and in area MT (t(22)=4.77, p=.000) but not in the OFA and FFA (p>.3), further supporting the sensitivity to motion in the dorsal but not the ventral face areas, as shown in the univariate results reported above. The same analysis was conducted using varying ROI volumes (30–80 voxels) to verify that the effect is not limited to a specific ROI size. The pSTS-FA, IFG-FA and area MT showed higher correlation within than between presentation types across the range of ROI sizes (pSTS-FA: p<.0004 for all ROI sizes; IFG-FA: p<.04 for all ROI sizes; MT: p<.02 for 30, 40, 50 and 60 voxels, p<.08 for 70 and 80 voxels). We then compared the correlations within and between Tasks. None of the ROIs showed higher correlation within than between tasks (p>.2) (see Supplementary Figure 4). Thus, the sensitivity to changeable facial aspects in the pSTS-FA in the univariate analysis is not apparent in the multi-voxel pattern of the neural response of these areas.

To assess whether the sensitivity to motion based on pattern analysis in the dorsal and ventral face areas is significantly different, we performed t-tests to directly compare data from the pSTS-FA and FFA. We found that the sensitivity to motion was significantly larger in pSTS-FA than in the FFA (t(20)=3.07, p=.006). The two ventral face areas, OFA and FFA, were similarly insensitive to motion (p>.6), and the two dorsal face areas, pSTS-FA and IFG-FA, were similarly sensitive to motion (p>.4).

References

1. Fox, C. J., Iaria, G. & Barton, J. J. S. Defining the face processing network: Optimization of the functional localizer in fMRI. *Hum. Brain Mapp.* **30,** 1637–1651 (2009).

2. Huk, A. C., Dougherty, R. F. & Heeger, D. J. Retinotopy and functional subdivision of human areas MT and MST. *J. Neurosci.* **22,** 7195–7205 (2002).

3. Brett, M., Anton, J.-L. L., Valabregue, R. & Poline, J.-B. Region of interest analysis using an SPM toolbox - Abstract Presented at the 8th International Conference on Functional Mapping of the Human Brain, June 2-6, 2002, Sendai, Japan. *Neuroimage* **16,** Abstract 497 (2002).

4. Kolster, H., Peeters, R. & Orban, G. A. The Retinotopic Organization of the Human Middle Temporal Area MT/V5 and Its Cortical Neighbors. *J. Neurosci.* **30,** 9801–9820 (2010).

5. Hastie, T., Tibshirani, R. & Friedman, J. *The Elements of Statistical Learning: Data Mining, Inference, and Prediction*. *Springer* (2009). doi:10.1007/b94608

6. Whitfield-Gabrieli, S. & Nieto-Castanon, A. *Conn* : A Functional Connectivity Toolbox for Correlated and Anticorrelated Brain Networks. *Brain Connect.* **2,** 125–141 (2012).

7. Blank, I., Kanwisher, N. & Fedorenko, E. A functional dissociation between language and multiple-demand systems revealed in patterns of BOLD signal fluctuations. *J. Neurophysiol.* (2014). doi:10.1152/jn.00884.2013

8. Kanwisher, N. & Yovel, G. The fusiform face area: a cortical region specialized for the perception of faces. *Philos. Trans. R. Soc. B Biol. Sci.* **361,** 2109–2128 (2006).

9. Haxby, J. V. Multivariate pattern analysis of fMRI: The early beginnings. *NeuroImage* **62,** 852–855 (2012).

10. Haxby, J. V. *et al.* Distrubuted and Overlapping Representations of Face and Objects in Ventral Temporal Cortex. *Science (80-. ).* **293,** 2425–2430 (2001).

11. Weiner, K. S. & Grill-Spector, K. Not one extrastriate body area: Using anatomical landmarks, hMT+, and visual field maps to parcellate limb-selective activations in human lateral occipitotemporal cortex. *Neuroimage* **56,** 2183–2199 (2011).

12. Krumhuber, E. G., Kappas, A. & Manstead, A. S. R. Effects of Dynamic Aspects of Facial Expressions: A Review. *Emot. Rev.* (2013). doi:10.1177/1754073912451349

**Supplementary Table 1**

The volume (averaged number of voxels and standard deviation) of the functionally defined ROIs and the number of participants^[[1]](#footnote-1)^ in which each of these areas was found.

|  |  | FFA | OFA | pSTS-FA | MT | IFG-FA |
| --- | --- | --- | --- | --- | --- | --- |
| Version 1 – Multi-static Ordered | Right | 171 (97) (n=17) | 221 (134) (n=10) | 490 (368) (n=17) | 134 (94) (n=13) | 90 (87) (n=5) |
|  | Left | 86 (65) (n=16) | 169 (231) (n=12) | 324 (402) (n=17) | 130 (104) (n=13) | 39 (30) (n=4) |
| Version 2 – Multi-static Scrambled | Right | 142 (157) (n=16) | 217 (145) (n=14) | 487 (371) (n=16) | 206 (155) (n=15) | 72 (94) (n=6) |
|  | Left | 105 (133) (n=13) | 125 (69) (n=14) | 336 (280) (n=14) | 193 (143) (n=15) | 22 (17) (n=2) |

**Supplementary Table 2**

Mean and standard deviation of accuracy (percent correct) and reaction time in the gender and expression tasks for the dynamic and the static faces in Versions 1 and 2.

| Version 1 |  | Static Faces  (in order) | Dynamic Faces |
| --- | --- | --- | --- |
| Accuracy | Gender Task | 87.8 (8.6) | 90.1 (11.0) |
|  | Expression Task | 81.7 (12.0) | 81.5 (12.0) |
| Reaction time | Gender Task | 2.53 (1.34) | 2.54 (1.23) |
|  | Expression Task | 3.32 (0.99) | 3.12 (1.03) |
| Version 2 |  | Static Faces  (Scrambled order) | Dynamic Faces |
| Accuracy | Gender Task | 90.3 (8.5) | 83.2 (8.1) |
|  | Expression Task | 83.2 (11.2) | 88.7 (6.7) |
| Reaction time | Gender Task | 1.26 (0.26) | 1.75 (0.26) |
|  | Expression Task | 1.75 (0.43) | 1.85 (0.35) |

**Supplementary Table 3**

Percent signal change (and standard error of the mean) in each ROI for dynamic and static faces during the expression and gender tasks, in Versions 1 and 2 and the number of subjects (N) that are included in the analysis for each ROI.

| IFG-FA | | pSTS-FA | | FFA | | OFA | | MT | |  | |
| --- | --- | --- | --- | --- | --- | --- | --- | --- | --- | --- | --- |
| Dyn | Stat | Dyn | Stat | Dyn | Stat | Dyn | Stat | Dyn | Stat |  |  |
| 0.41 (0.16) | 0.43 (0.14) | 0.51 (0.07) | 0.36 (0.06) | 1.02 (0.07) | 1.00 (0.07) | 0.83 (0.09) | 0.90 (0.12) | 0.93 (0.16) | 0.62 (0.15) | Gender | Ver. 1 |
| 0.49 (0.10) | 0.32 (0.11) | 0.68 (0.07) | 0.55 (0.08) | 1.10 (0.08) | 1.12 (0.08) | 1.02 (0.07) | 1.05 (0.11) | 1.03 (0.23) | 0.76 (0.19) | Expression |  |
| 5 | | 17 | | 17 | | 14 | | 14 | | N |  |
| 0.84 (0.17) | 0.31 (0.12) | 0.49 (0.08) | 0.30 (0.08) | 1.16 (0.13) | 1.13 (0.16) | 0.99 (0.10) | 0.98 (0.10) | 1.07 (0.13) | 0.71 (0.11) | Gender | Ver. 2 |
| 1.12 (0.21) | 0.85 (0.31) | 0.68 (0.08) | 0.50 (0.09) | 1.22 (0.15) | 1.20 (0.12) | 0.94 (0.12) | 0.96 (0.14) | 1.16 (0.17) | 0.82 (0.15) | Expression |  |
| 6 | | 16 | | 17 | | 17 | | 16 | | N |  |

Figure Legends

**Supplementary Figure 1: Example Stimuli.** Faces were cropped to show only the internal facial features, with no hair or other external facial features. The dynamic conditions included 4 second movie-clips of neutral faces turning happy (a) or disgust (b). The static conditions included four images from the movie (the first frame of each second) presented for 1 second each.

**Supplementary Figure 2: ROI localization.** a. Dynamic face-selective areas were defined as the contrast between Dynamic Faces > Dynamic Objects, and the motion-selective area MT was defined as the contrast between Dynamic Rings > Static Rings. b. fMRI responses in area MT were similar to the dynamic faces and objects used to localize the face areas. Error bars indicate the standard error of the mean difference between dynamic faces and dynamic objects and are therefore displayed on one of the bars.

**Supplementary Figure 3: Face and motion selective areas.** Lateral (a) and ventral (b) views of the right hemisphere of representative subjects showing the face areas, OFA, FFA, pSTS-FA and IFG-FA in red and area MT in blue.

**Supplementary Figure 4: Multivariate analysis results.** Pattern similarity analysis suggests sensitivity to motion in the pSTS-FA, IFG-FA and area MT but not in the OFA and FFA. Error bars indicate the standard error of the correlation difference ** p < .01; *** p<.001.

**Supplementary Figure 1**

**
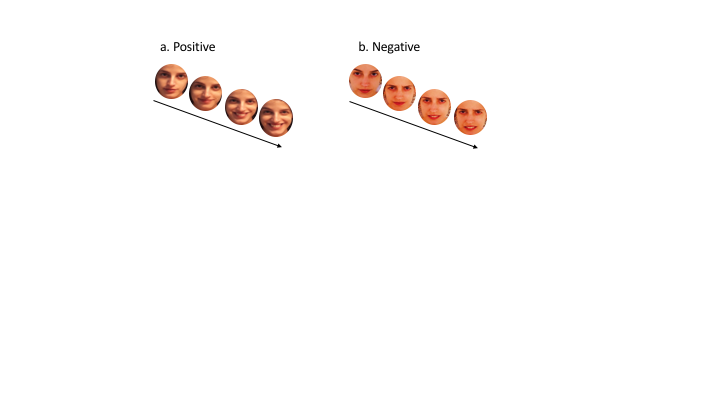
**

**Supplementary Figure 2**


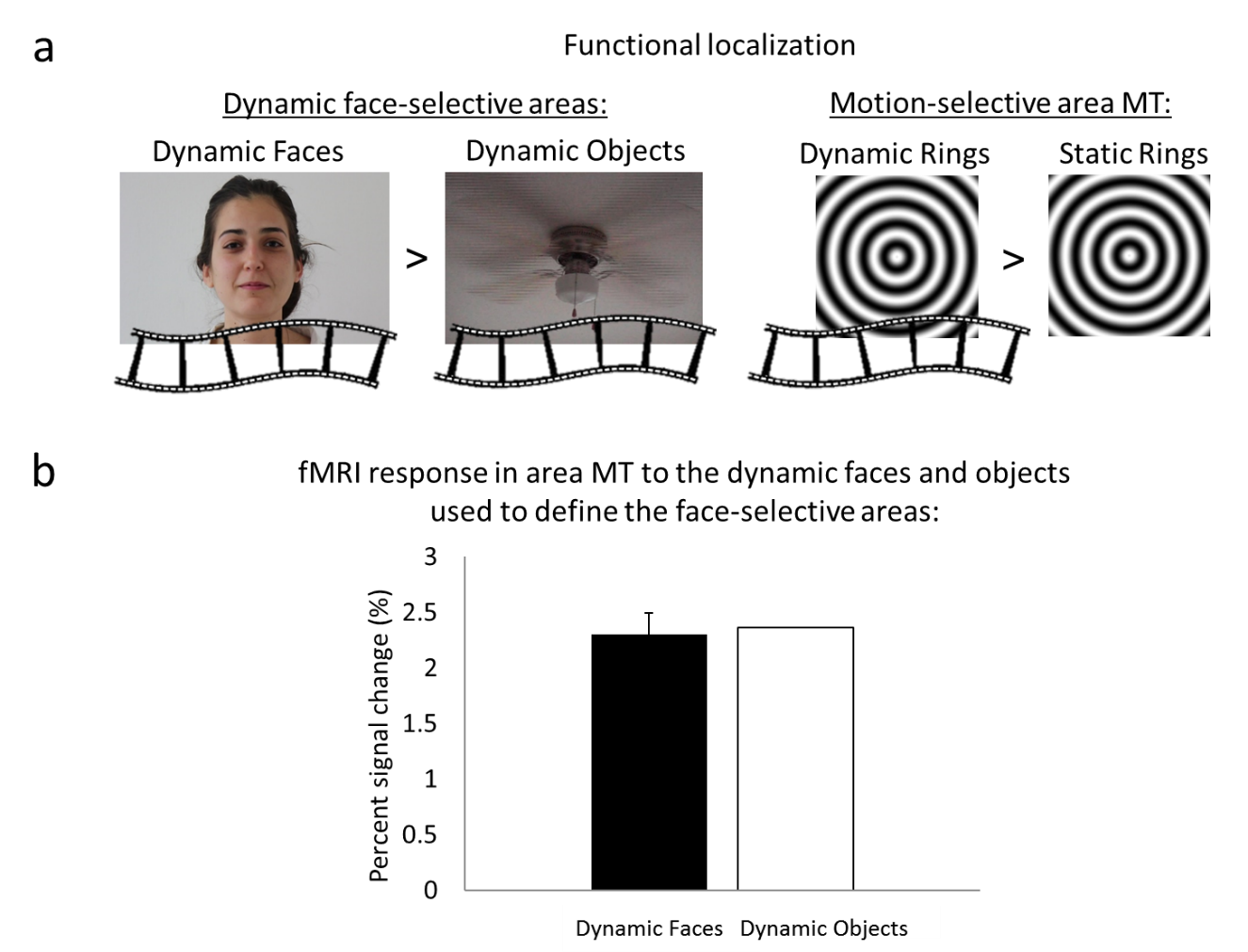


**Supplementary Figure 3**


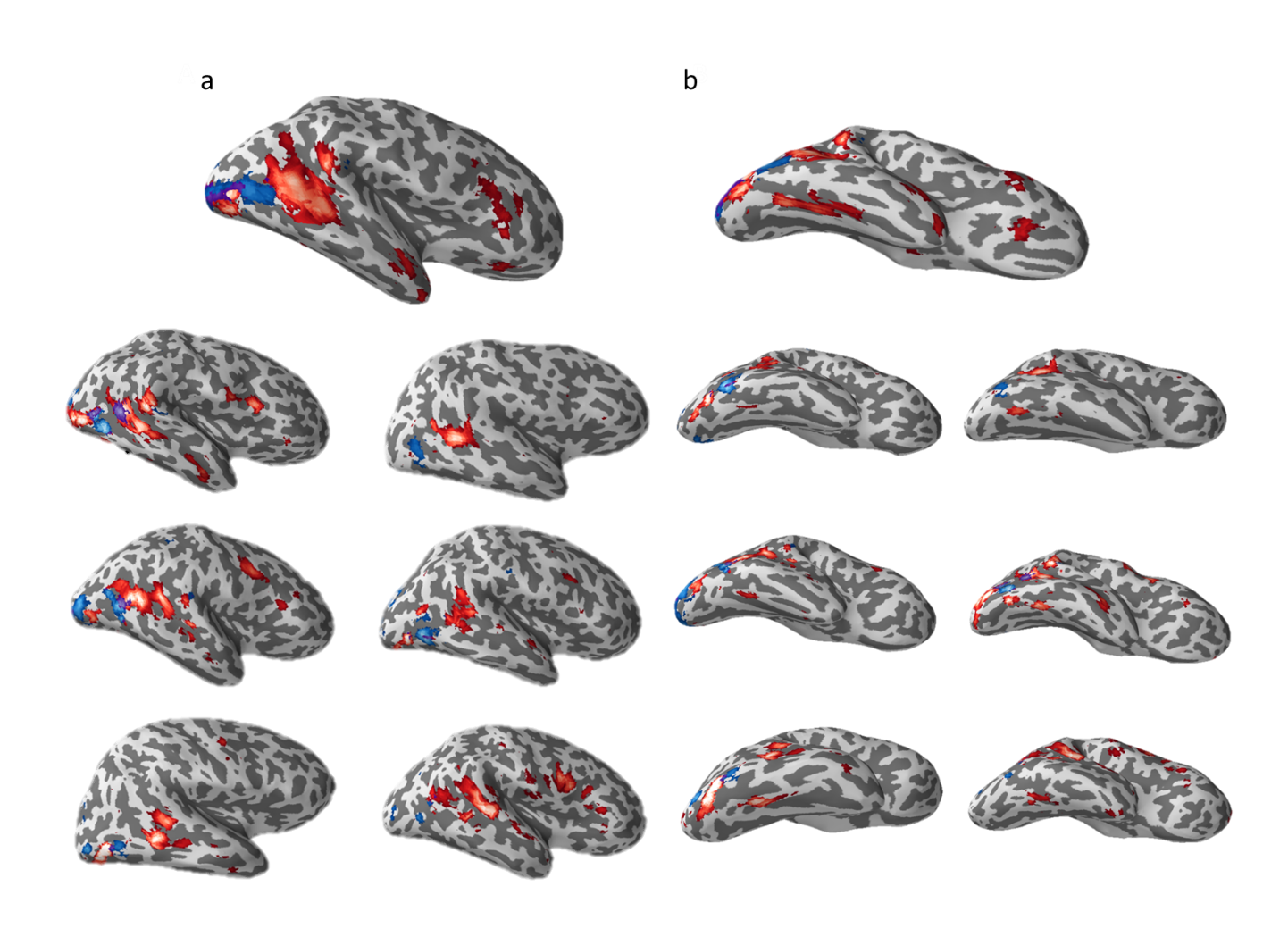


**Supplementary Figure 4**


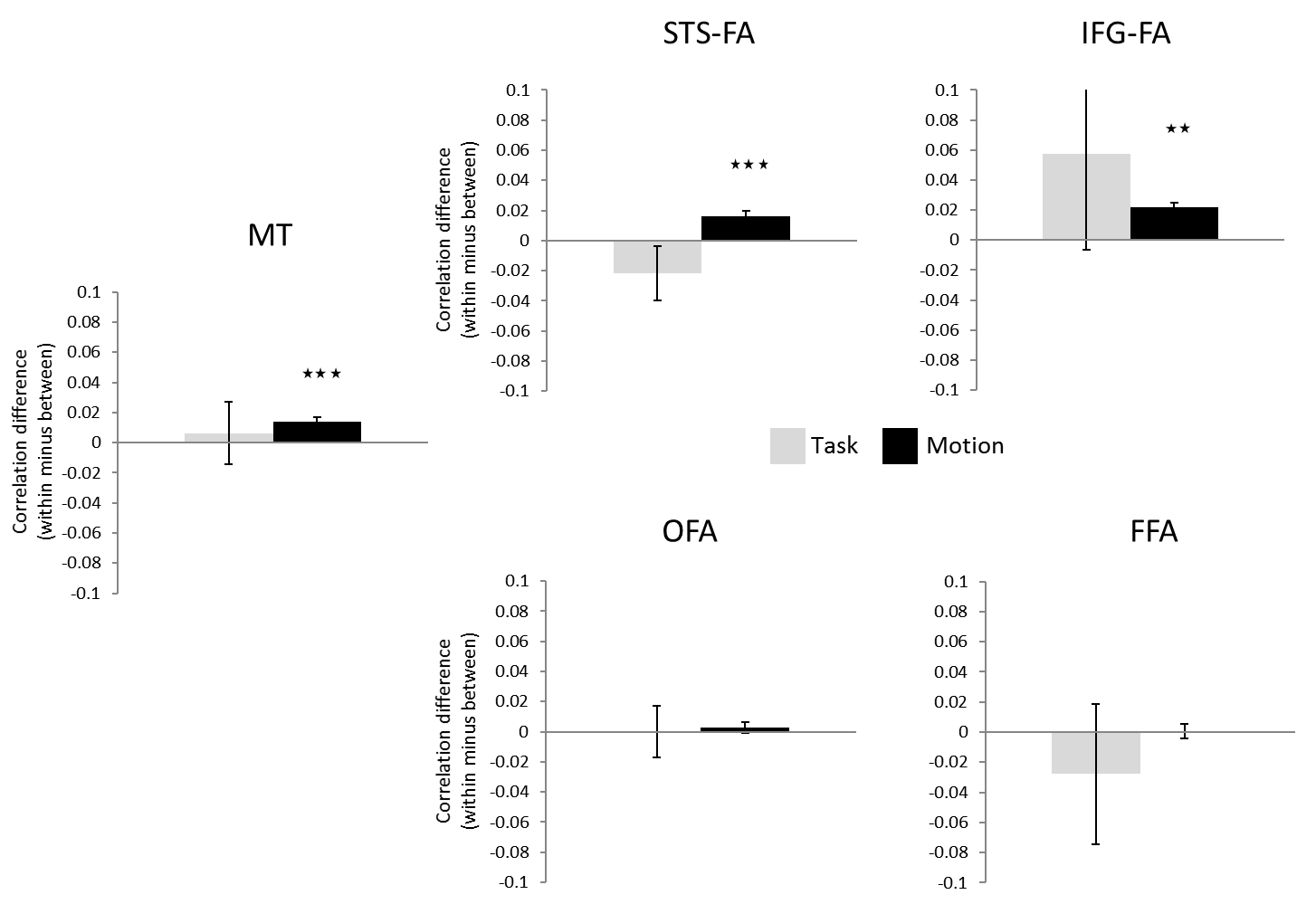


1. Note that the number of subjects in each ROI may not reflect the number of subjects that included in the analysis in which data from the two hemispheres were averaged (see Data Analysis). For example, for the OFA in Version 1, the table indicates 10 and 12 subjects in right and left OFA, respectively, but overall 14 subjects were included in the analysis because 8 subjects showed activations in both hemispheres, 2 subjects showed only right hemisphere activation and 4 subjects showed only left hemisphere activations resulting in 14 subjects across both hemispheres. [↑](#footnote-ref-1)
